# Soluble Aβ_1–42_ increases the heterogeneity in synaptic vesicle pool size among synapses by suppressing intersynaptic vesicle sharing

**DOI:** 10.1186/s13041-018-0353-z

**Published:** 2018-02-20

**Authors:** Daehun Park, Sunghoe Chang

**Affiliations:** 1 Department of Physiology and Biomedical Sciences, Seoul National University College of Medicine, Seoul, 03080 South Korea; 2 Neuroscience Research Institute, Medical Research Center, Seoul National University College of Medicine, Seoul, 03080 South Korea

## Abstract

Growing evidence has indicated that prefibrillar form of soluble amyloid beta (sAβ_1–42_) is the major causative factor in the synaptic dysfunction associated with AD. The molecular changes leading to presynaptic dysfunction caused by sAβ_1–42_, however, still remains elusive. Recently, we found that sAβ_1–42_ inhibits chemically induced long-term potentiation-induced synaptogenesis by suppressing the intersynaptic vesicle trafficking through calcium (Ca^2+^) dependent hyperphosphorylation of synapsin and CaMKIV. However, it is still unclear how sAβ_1–42_ increases intracellular Ca^2+^ that induces hyperphosphorylation of CaMKIV and synapsin, and what is the functional consequences of sAβ_1–42_-induced defects in intersynaptic vesicle trafficking in physiological conditions. In this study, we showed that sAβ_1–42_elevated intracellular Ca^2+^ through not only extracellular Ca^2+^ influx but also Ca^2+^ release from mitochondria. Surprisingly, without Ca^2+^ release from mitochondria, sAβ_1–42_ failed to increase intracellular Ca^2+^ even in the presence of normal extracellular Ca^2+^. We further found that sAβ_1–42_-induced mitochondria Ca^2+^ release alone sufficiently increased Serine 9 phosphorylation of synapsin. By blocking synaptic vesicle reallocation, sAβ_1–42_ significantly increased heterogeneity of total synaptic vesicle pool size among synapses. Together, our results suggested that by disrupting the axonal vesicle trafficking, sAβ_1–42_ disabled neurons to adjust synaptic pool sizes among synapses, which might prevent homeostatic rescaling in synaptic strength of individual neurons.

**Electronic supplementary material:**

The online version of this article (10.1186/s13041-018-0353-z) contains supplementary material, which is available to authorized users.

## Background

Abnormal synaptic function is one of the earliest known defects in Alzheimer’s disease (AD) [1]. Recent studies have indicated that the non-fibrillar soluble oligomeric form of amyloid β protein (sAβ) rather than insoluble amyloid fibrils or plaques [2–4] is the cause of the synaptic dysfunction and cognitive defects associated with AD. Indeed, biochemical analysis of postmortem AD tissue has revealed a robust correlation between sAβ levels and the extent of synapse loss and cognitive impairment [2]. The accumulation of sAβ also closely correlates with cognitive decline in animal models and AD patients and is primarily due to disrupting synaptic plasticity [5], Ca^2+^ homeostasis [6–8] and signaling pathways such as glycogen synthase kinase 3 beta (GSK-3β) [9], c-Jun [10], Ca^2+^/calmodulin-dependent protein kinase kinase (CaMKK), AMP-activated protein kinase (AMPK) [11], cytoskeletal networks [12] and axonal transport [13]. The 42-residue amyloid beta protein (sAβ_1–42_) has been shown to impair long-term potentiation (LTP), and to be neurotoxic [14]. A number of different postsynaptic mechanisms, including dendritic spine loss, alteration of 2-amino-3-(5-methyl-3-oxo-1,2-oxazol-4-yl) propanoic acid (AMPA) and N-methyl-D-aspartic acid (NMDA) receptor numbers have been implicated in sAβ_1–42_-induced synaptic dysfunction [15–17] while the molecular changes leading to presynaptic dysfunction by sAβ_1–42_ have not been clearly identified [18–23].

Previous studies have showed that axonal synaptic vesicles diffuse laterally along the axon and trading of synaptic vesicles (SVs) between synapses reallocates functional SV pools and synaptic strength, leading to dynamically regulate presynaptic properties [24–26]. One of physiological consequences of intersynaptic vesicle sharing includes a rapid new functional synapse formation upon synaptic plasticity [26, 27]. Recently, we have found that sAβ_1–42_ inhibits chemical LTP (cLTP)-induced synaptogenesis by suppressing the intersynaptic vesicle trafficking. We further found that sAβ_1–42_ rapidly increases intracellular Ca^2+^, which causes hyperphosphorylation of synapsin and CaMKIV and this is a key pathway responsible for the inhibitory effect of sAβ_1–42_ on the regulation of intersynaptic vesicle trafficking [27]. We, however, do not know how sAβ_1–42_ increases intracellular Ca^2+^ which is critical for the phosphorylation-dependent dissociation of synapsin-SV-actin ternary complex [27]. The sharing between the SV pools over synapse also contributes to resizing of the SV pool at a single synapse, leading to homeostatic changes in synaptic pool sizes in neurons [26]. Since sAβ_1–42_suppresses intersynaptic vesicle trafficking, it could affect homeostatic regulation of SV pool size, which is currently unknown.

In this study, we have addressed these two important issues. We found that sAβ_1–42_ rapidly elevated intracellular Ca^2+^ through not only extracellular Ca^2+^ influx but also Ca^2+^ release from mitochondria. Surprisingly, sAβ_1–42_ induced Ca^2+^ release from mitochondria is critical for extracellular Ca^2+^ influx, and it also sufficiently hyperphosphorylates synapsin which is important for intersynaptic vesicle trafficking. We also showed that acute treatment of sAβ_1–42_ to cultured rat hippocampal neurons strongly blocked SV reallocation, leading to a significant increase in heterogeneity in SV pool size among synapses.

## Results

### sAβ_1–42_-induced Ca^2+^ release from mitochondria is critical for extracellular Ca^2+^ influx

**Fig. 1:**
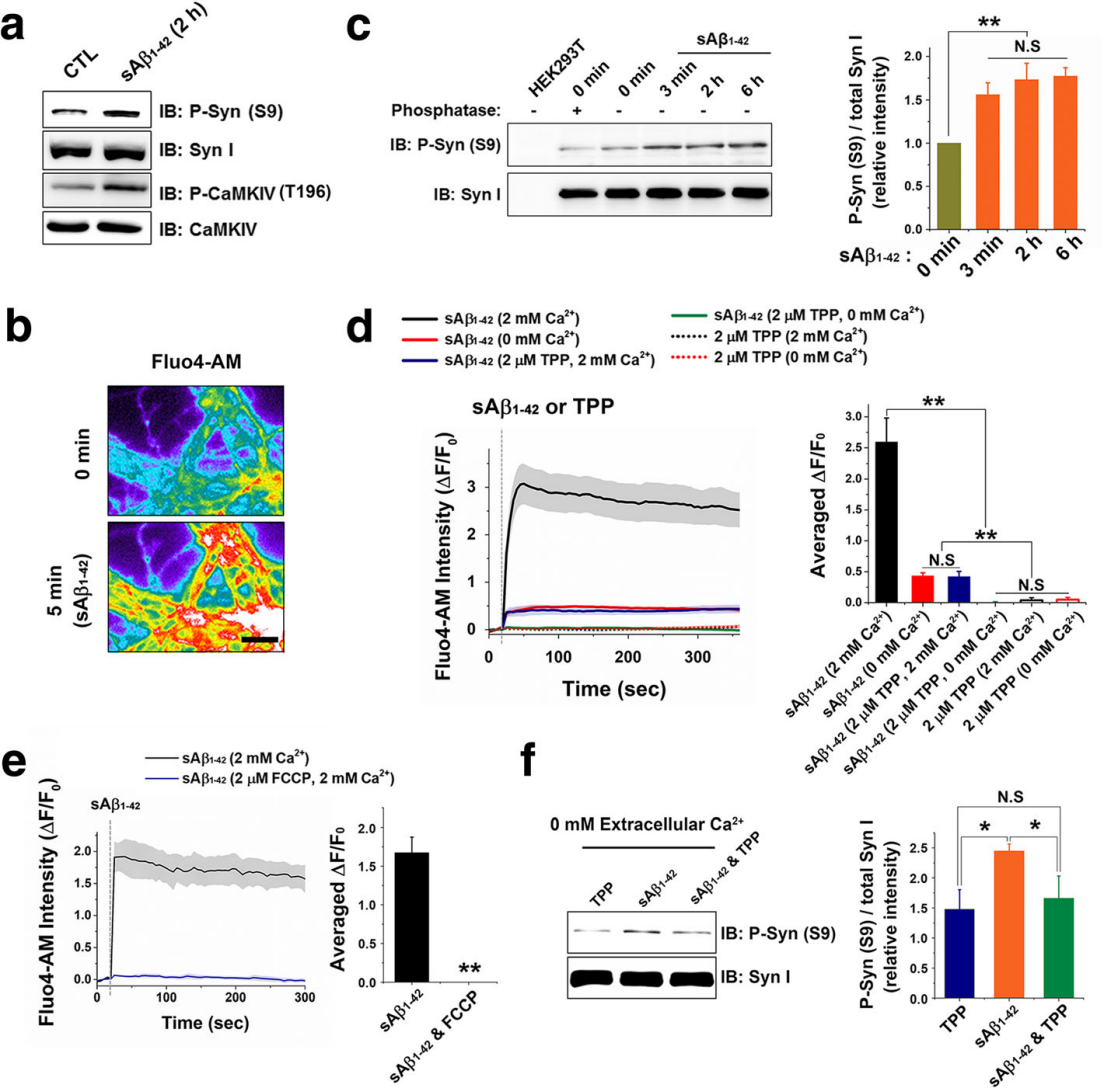
sAβ_1–42_ increases cytosolic Ca^2+^ concentration by inducing mitochondria Ca^2+^ release dependent extracellular Ca^2+^ influx. **a** Cultured neurons were pretreated as indicated and analyzed for the level of each phospho-synapsin (S9) and phospho-CaMKIV (T196). **b** Representative images for Fluo-4 AM after treatment with 200 nM sAβ_1–42_ for 5 min (Scale bar = 5 μm). **c** Neurons were treated with sAβ_1–42_ for the time as indicated and phosphorylation of synapsin was measured by the western blot (1 ± 0 for 0 min, 1.56 ± 0.13 for 3 min, 1.73 ± 0.19 for 2 h, 1.77 ± 0.10 for 6 h sAβ_1–42_ treatment, *n* = 4 independent blots). Equal amount of HEK293T cell lysates and neuron lysates incubated with phosphatase were loaded to confirm the synapsin and phospho-synapsin bands. **d** Fluo-4 AM intensity plots after treatment with 200 nM sAβ_1–42_ or 2 μM TPP in the indicated extracellular buffer (in brackets). Averaged ΔF/F_0_ was calculated by averaging the last 20 points of fluorescence profiles (2.59 ± 0.39 for sAβ_1–42_ in 2 mM extracellular Ca^2+^, 0.43 ± 0.05 for sAβ_1–42_ in 0 mM extracellular Ca^2+^, 0.42 ± 0.08 for sAβ_1–42_ in 2 mM extracellular Ca^2+^ with TPP, 0.00 ± 0.01 for sAβ_1–42_ in 0 mM extracellular Ca^2+^ with TPP, 0.04 ± 0.04 for TPP in 2 mM extracellular Ca^2+^, 0.05 ± 0.03 for TPP in 0 mM extracellular Ca^2+^ (*n* ≥ 5 independent experiments for each group)). **e** Fluo-4 AM loaded neurons were pretreated as indicated (in brackets) for 5 min and treated with 200 nM sAβ_1–42_ (Averaged ΔF/F_0_: 1.67 ± 0.20 for sAβ_1–42_ (*n* = 3 independent experiments), 0.00 ± 0.03 for sAβ_1–42_ with FCCP (*n* = 4 independent experiments)). **f** Neurons were treated with the indicated medium for 2 h and their phospho-synapsin (S9) and total synapsin level were detected by western blot and analyzed (Phospho-synapsin/total-synapsin: 1 ± 0 for control, 1.48 ± 0.32 for control with TPP, 2.45 ± 0.11 for sAβ_1–42_, 1.66 ± 0.37 for sAβ_1–42_ with TPP, *n* = 3 independent blots). Values are means ± standard error of mean (SEM). N.S = no significant difference, * *p* < 0.05, ** *p* < 0.01 (ANOVA and Tukey’s HSD post hoc test for (**c**, **d**, **f**) and Student’s *t*-test for (**e**))

Our previous study have found that acute treatment (2 h) of sAβ_1–42_ greatly increases the presynaptic Ca^2+^ level, which leads to hyperphosphorylation of CaMKIV (T196) and synapsin (S9) [27] (also confirmed in Fig. 1a). Ca^2+^ and CaMKIV mediated phosphorylation of synapsin S9 dissociates SV-synapsin-actin ternary complex and this is a critical pathway for sAβ_1–42_ effect to inhibit intersynaptic vesicle trafficking [27]. However, the source of cytosolic Ca^2+^ upraised by sAβ_1–42_ treatment has remained unknown. To solve this question, we loaded neurons with Fluo-4 AM to monitor changes in cytosolic Ca^2+^ levels (Fig. 1b). sAβ_1–42_ markedly increased intracellular Ca^2+^ after 5 min treatment in 2 mM extracellular Ca^2+^ concentration (Fig. 1b). Similar to this result, phosphorylation of synapsin was also increased after sAβ_1–42_ treatment (Fig. 1c). The amount of phosphorylated synapsin by sAβ_1–42_ was not affected by treatment time (3 min to 6 h) (Fig. 1c). Next, we measured sAβ_1–42_-induced cytosolic Ca^2+^ elevation in real-time and found that sAβ_1–42_ rapidly increases intracellular Ca^2+^ right after treatment in 2 mM extracellular Ca^2+^ concentration (Fig. 1d). sAβ_1–42_, however, also evoked a small but significant rises cytosolic Ca^2+^ even in the absence of extracellular Ca^2+^ (Fig. 1d). These results suggested that sAβ_1–42_ increased the cytosolic Ca^2+^ level by mainly inducing extracellular Ca^2+^ influx but partially stimulating the other intracellular Ca^2+^ stores. Mitochondria have been known to act as important internal Ca^2+^ source and to be dysregulated in Alzheimer’s disease [28]. To test the effects of sAβ_1–42_ on mitochondria Ca^2+^ release, we blocked mitochondrial Ca^2+^ efflux by applying tetraphenylphosphonium (TPP), which blocks Ca^2+^ efflux from mitochondria [29]. TPP completely eliminated sAβ_1–42_-induced rise in Ca^2+^signal in the absence of extracellular Ca^2+^ (Fig. 1d). Surprisingly, when mitochondria Ca^2+^ efflux was blocked by TPP, cytosolic Ca^2+^ increment by sAβ_1–42_ was significantly decreased despite the presence of 2 mM extracellular Ca^2+^ (Fig. 1d). Furthermore, when we pretreated carbonyl cyanide p-(trifluoromethoxy)-phenylhydrazone (FCCP) to deplete the mitochondria Ca^2+^ and then treated with sAβ_1–42_ (Fig. 1e), the increment of cytosolic Ca^2+^ by sAβ_1–42_ was not observed even in the presence of extracellular Ca^2+^ (Fig. 1e).

Next we tested whether mitochondrial Ca^2+^ release by sAβ_1–42_ is sufficient to phosphorylate synapsin. We found that phosphorylation of synapsin was signicantly increased by sAβ_1–42_-induced mitochondrial Ca^2+^release (without extracellular Ca^2+^) and restored by TPP treatment (Fig. 1f). These results indicated that sAβ_1–42_-evoked rise in cytosolic Ca^2+^ was mostly due to the Ca^2+^ coming from outside of the cells, but the release of Ca^2+^ from the mitochondria plays an important role to induce extracellular Ca^2+^ influx and could phosphorylates synapsin.

### sAβ_1–42_ inhibits intersynaptic movements of synaptic vesicle and synapsin

**Fig. 2:**
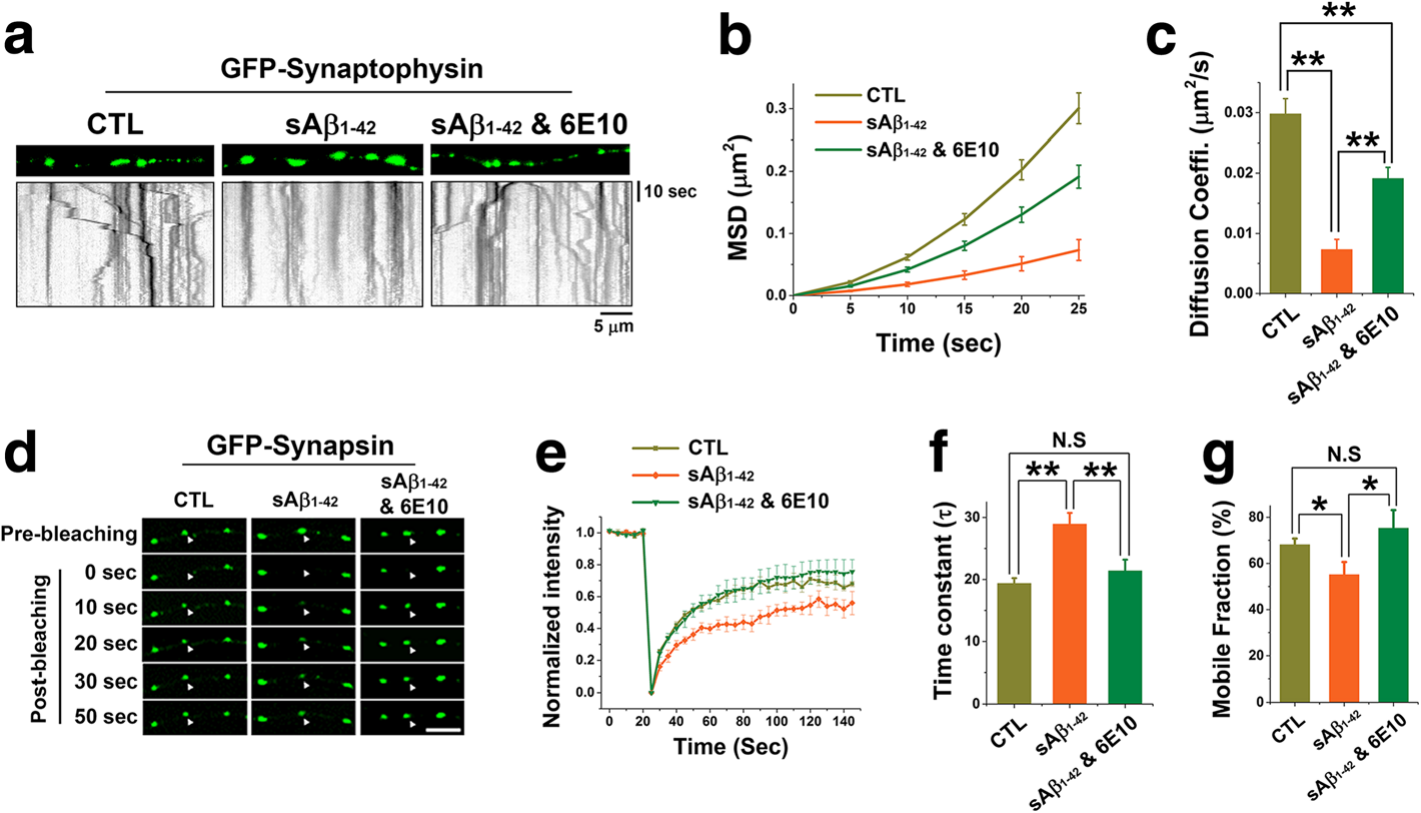
sAβ_1–42_ suppressed the intersynaptic vesicle movement and synapsin. **a-c** Neurons expressing GFP-synaptophysin were treated as indicated and imaged for 1 min to track the intersynaptic vesicles. **a** Representative kymographs showing trafficking of GFP-synaptophysin in each group. **b** MSD curves versus time. **c** Diffusion coefficients, *n* = 7 independent experiments for each group. **d-g** Neurons were transfected with GFP-synapsin (DIV8) and treated as indicated at DIV14. A single bouton was selectively photobleached and its recovery time was measured through FRAP assay. **d** Representative time-lapse images of FRAP assay for control, sAβ_1–42_ and sAβ_1–42_ with 6E10 (scale bar = 5 μm). White arrowheads indicate bleached boutons. **e** Average plots of fluorescence intensities (normalized to initial intensities) before and after photobleaching. **f** Fluorescence recovery traces in (**e**) were fitted to a single exponential function with time constant (τ) of 19.36±0.84 s for control, 28.92±1.8 s for sAβ_1–42_, and 21.38±1.82 s for sAβ_1–42_ with 6E10, respectively (*n* = 4 independent experiments for each group). **g** Mobile fractions were calculated by averaging final 5 values in (**e**). Mobile fraction (%): 68.10±2.67 for control, 55.13±5.47 for sAβ_1–42_, and 75.25±7.85 for sAβ_1–42_ with 6E10 (*n* = 4 independent experiments for each group). Values are means±SEM. N.S = no significant difference, * *p* < 0.05, ** *p* < 0.01 (ANOVA and Tukey’s HSD post hoc test))

Since phosphorylation of synapsin is a key mechanism for sAβ_1–42_-induced defect in the intersynaptic vesicle trafficking, the overexpression of phospho-deficient mutant of synapsin Ia completely restore the sAβ_1–42_-induced inhibition of intersynaptic vesicle movements [27]. Accordingly, we further found that phospho-deficient mutant (S9A) of synapsin Ia had much higher binding affinity for actin, which is important for maintaining intersynaptic trafficking (Additional file 1: Figure S1). In addition, we confirmed that sAβ_1–42_ strongly suppressed intersynaptic vesicle trafficking as previously described (Fig. 2a-c) [27].

Previous study showed that synapsin itself laterally moves between neighboring synapses [30]. Thus, we examine the dynamic behavior of synapsin in response to sAβ_1–42_ using a fluorescence recovery after photobleaching (FRAP) and analyzed whether sAβ_1–42_ affects the movement of synapsin as well. We transfected GFP-synapsin into neurons, and then selectively photo-bleached a single presynapse and monitored the recovery of fluorescence (Fig. 2d). After photo-bleaching, substantial recovery of GFP-synapsin fluorescence (~ 70%) was observed in the control neurons (Fig. 2d, e). However, in boutons treated with sAβ_1–42_, fluorescence recovery occurred less and slower than in the control group (Fig. 2d-g). Preincubation of sAβ_1–42_ with 6E10 antibody completely blocked the sAβ_1–42_ effect (Fig. 2d-g). These data strongly suggested that sAβ_1–42_ suppressed the intersynaptic movements of both SVs and synapsin.

### sAβ_1–42_ significantly increases heterogeneity of total SV pools among synapses

Since the lateral trafficking and sharing of SVs among synapses have been known to regulate presynaptic properties by reallocating SVs and thus balancing the SV pool size among synaptic neighbors [24, 26, 31], we suspected that the defects in lateral intersynaptic trafficking of SV by sAβ_1–42_ could affect homeostatic regulation of SV pool size among synapses.

**Fig. 3:**
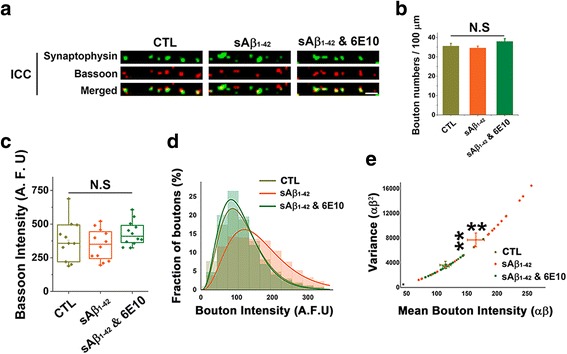
sAβ_1–42_ increases heterogeneity of presynaptic terminals. **a-e** Neurons were treated as indicated and immunostained for endogenous synaptophysin and bassoon. **a** Representative immunocytochemistry images (scale bar = 2 μm). **b** The number of presynaptic boutons were counted along the axon (Average bouton numbers per 100 μm axon length: 35.54±1.43 for control, 34.57±0.97 for sAβ_1–42_, and 37.92±1.53 for sAβ_1–42_ with 6E10 (*n* = 4 independent experiments for each group)). **c** The intensity of bassoon puncta (> 80,000) was measured and presented with the box plot. **d** The intensity profiles of synaptophysin-stained boutons were fitted into gamma distribution with a fixed shape parameter α = 4. The scale parameter β was as follows: Control = 29.56 (81,356 boutons); sAβ_1–42_ = 40.70 (89,599 boutons); sAβ_1–42_ with 6E10 = 28.20 (83,369 boutons). **e** Raw data of (d) were fitted to gamma function and their mean bouton intensity (α x β) and variance (α x β^2^) were measured. Values are means±SEM. N.S = no significant difference, ** *p* < 0.01 (ANOVA and Tukey’s HSD post hoc test)

We first stained neurons with the synaptic vesicle marker, synaptophysin and the active zone marker, bassoon after treatment with or without sAβ_1–42_ to check if there are any morphological changes in presynaptic terminals. When we stained neurons with synaptophysin antibody to measure total synaptic vesicle pool size [32–34], we found that sAβ_1–42_ treated neurons showed higher variability of the size of presynaptic boutons than the control group, while they showed the similar number of presynaptic boutons with the control group (Fig. 3a, b). The intensity of bassoon, a presynaptic active zone marker, however, was not different between sAβ_1–42_ treated and untreated neurons (Fig. 3c), demonstrating that sAβ_1–42_ treatment increased heterogeneity of total SV pool size without altering the morphology of presynaptic terminals.

To confirm that this did not come from a biased undersampling, we plotted the pooled distribution histogram of synaptophysin intensities obtained from over 80,000 individual boutons. We found that sAβ_1–42_ increased the mean value of the total SV pool size (Fig. 3d, e). More importantly, when the distribution of bouton intensities was fitted to a gamma function with a fixed-shape parameter (α = 4), the scale parameter (β), which indicates the degree of dispersion of the distribution, was significantly larger in neurons exposed to sAβ_1–42_ than in the control or sAβ_1–42_-6E10 treated group (Fig. 3d, e). These results indicate that sAβ_1–42_ significantly increases heterogeneity between presynapses and thus affects homeostatic rescaling by inhibiting intersynaptic vesicle trafficking.

## Discussion

Substantial data have indicated that sAβ_1–42_ causes the synaptic dysfunction observed in AD [3]. sAβ_1–42_ alters synaptic plasticity by inhibiting long-term potentiation and facilitating long-term depression [35, 36]. These changes induce the loss of dendritic spines, modulate the expression of AMPA and NMDA receptors and interfere with Ca^2+^ homeostasis [4, 15, 17, 37]. Much less attention, however, has been paid to the effect of sAβ_1–42_ on the presynaptic function. Moreover, there have been highly contradictory observations about the effects of sAβ_1–42_ on presynapses depending on its source [18, 20] or concentration [18, 22]. Here, we found that sAβ_1–42_ induces mitochondrial Ca^2+^ release and it is critical for extracellular Ca^2+^ influx across the plasma membrane and hyperphosphorylation of synapsin. We also have figured out that sAβ_1–42_ strongly increases heterogeniety of presynaptic vesicle pool sizes by disrupting SV pool sharing which is affected by synapsin phosphorylation.

We also have found that sAβ_1–42_-mediated Ca^2+^ increment is the major causative factor in the presynaptic dysfunction associated with intersynaptic vesicle trafficking [27]. However, we have not revealed the Ca^2+^ sources involved in this phenomenon [27]. Although the precise mechanism is still elusive, previous studies have suggested that sAβ_1–42_ trigger not only internal Ca^2+^release from endoplasmic reticulum (ER) [38] but also extracellular Ca^2+^ influx by altering membrane Ca^2+^permeability, interacting with voltage-gated Ca^2+^channels or forming Aβ pores, [7, 39]. Here, we found that extracellular Ca^2+^ made up a large portion of the total Ca^2+^ incremented by sAβ_1–42_ treatment, whereas small but significant portion was constituted by Ca^2+^released from mitochondria. In addition, mitochondria Ca^2+^ efflux was required for the extracellular Ca^2+^ influx by sAβ_1–42_. Although the precise molecular mechanism of how released Ca^2+^ from mitochondria induces extracellular Ca^2+^ influx certainly requires further study, we could speculate some possibilities. The released Ca^2+^ from mitochondria by sAβ_1–42_ could act as a signaling molecule and activates some Ca^2+^ channels in plasma membrane. For example, previous study indicates that mitochondrial Ca^2+^ released by FCCP activates extracellular signal regulated kinase (ERK) 1/2 in PC12 cells [40]. Indeed, ERK phosphorylates α1 and β subunits of N-type VDCCs (voltage-dependent calcium channels) [41] and enhances VDCC current in sensory neurons [42]. Therefore, although the amount of Ca^2+^ released from mitochondria by sAβ_1–42_ is small, it may play critical role in the early stage of Ca^2+^ signaling. In this study, we also found that sAβ_1–42_-induced Ca^2+^ release from mitochondria without extracellular Ca^2+^ influx was sufficient to increase S9 phosphorylation of synapsin, indicating that small amount of Ca^2+^ release from mitochondria could sufficiently regulate the intersynaptic vesicle trafficking. However, unlike FCCP, TPP pretreatment did not completely block the sAβ_1–42_-induced Ca^2+^ influx across the membrane. These discrepancies may be due to the differences in the molecular mechanism of action, side effects of drugs or dose dependent manners. FCCP, a mitochondira proton gradient uncoupler, induces mitochondrial Ca^2+^ release as a proton inophore [43]. On the contrary, TPP specifically blocks both sodium-dependent and independent Ca^2+^ efflux from mitochondria [44]. However, TPP has around 50 times lower inhibitory constant (K_i_) value in sodium-dependent pathway than sodium-independent pathway and it means that sodium-dependent Ca^2+^ efflux is efficiently blocked by TPP [44]. In addition, previous study has showed that FCCP induces release of Ca^2+^ from not only mitochondria but also other non-mitochondrial Ca^2+^ sources [45]. Thus, we still cannot completely rule out the other internal Ca^2+^ sources for sAβ_1–42_-induced cytosolic Ca^2+^ elevation. Specifically, ER can have a significant role because ER and mitochondria work together to regulate intracellular Ca^2+^ levels [46]. In addition, previous study has shown that sAβ_1–42_ forms a cation-selective channels on the membrane and Zn^2+^ treatment can block the open pore [47]. Therefore, we tried to test the blockade effect of Zn^2+^ in the sAβ_1–42_-induced cytosolic Ca^2+^ elevation. However, preincubation of Zn^2 +^ largely increased basal Fluo-4 AM intensity (data not shown) and thus experiment could not proceed any further. However, our results strongly suggested that the mitochondrial Ca^2+^ release by sAβ_1–42_ plays an important role in cytosolic Ca^2+^ elevation and synapsin phosphorylation.

Although previous studies have showed that synaptic vesicles are shared constitutively between presynaptic terminals [24, 48], little is known about their functional roles and regulation mechanisms. One key aspect of vesicle sharing is its significant role in a variety of different forms of plasticity. We found that sAβ_1–42_ strongly inhibited activity-dependent rapid synaptogenesis, suggesting that inhibition of intersynaptic vesicle trafficking could be one of the cellular mechanisms underlying the sAβ_1–42_-induced defects in synaptic plasticity [49]. Conversely, this type of mechanism for allocating synaptic weights across multiple neighboring synapses could contribute to presynaptic homeostatic rescaling or balancing of SV pool size among synapses. In this study, we demonstrated that sAβ_1–42_ disrupted the regulatory mechanism of balancing SV pool sizes between presynaptic terminals by inhibiting intersynaptic vesicle sharing and thus increases the heterogeneity in SV pool size. In either case, the inhibition of intersynaptic trafficking caused by sAβ_1–42_ alters synaptic strength and efficacy, leading to the defects in synaptic plasticity and homeostatic regulation, which could contribute to synaptic dysfunctions observed in early AD.

Finally, although the in vitro system used in this study does not mimic the exact disease state that underlies AD, our results identify the novel sAβ_1–42_-induced defect in presynaptic function associated with the early stages of AD. Therefore, this work suggests a possible therapeutic target that prevents sAβ_1–42_-induced synaptic dysfunction in early-stage AD.

## Methods

### sAβ_1–42_ preparation and treatment

sAβ_1–42_ was prepared from synthetic Aβ_1–42_ peptide (Bachem) as previously described [27, 50, 51]. Briefly, 1 mM HFIP (1,1,1,3,3,3-hexafluoro-2-propanol, Sigma) was added to dissolve synthetic Aβ_1–42_ peptide and incubated at room temperature (RT) for 1 h. Then, HFIP was evaporated and dried from aliquots to make peptide film. Peptide film was dissolved in 1 mM dimethyl sulfoxide (DMSO) and Ham’s F-12 (phenol red-free, ThermoFisher scientific) was added for dilution and incubated over 12 h at 4 °C for oligomerization. sAβ_1–42_ oligomer was confirmed by western-blot before experiments. Unless otherwise indicated, prepared sAβ_1–42_ was diluted with cultured neurobasal media to the final concentration of 200 nM and treated to neurons for 2 h. To eliminate the sAβ_1–42_ effects, diluted sAβ_1–42_ was preincubated with Aβ antibody, 6E10 (Covance) for 2 h before treatment.

### Antibodies

Anti-bassoon (cat# ab82958, Abcam), anti-synaptophysin 1 (cat# 101011, Synaptic Systems), anti-phospho-S9-synapsin (cat# 2311, Cell Signaling Technology), anti-synapsin I (cat# 106103, Synaptic Systems), anti-phospho-Thr196-CaMKIV (cat# Sc-28,443-R, Santa Cruz Biotechnology), anti-CaMKIV (cat# ab3557, Abcam), anti-mCherry (cat# ab167453, Abcam), anti-actin (cat# A4700, Sigma) and 6E10 antibody (cat# SIG-39300, Covance) were used in the experiments.

### Hippocampal neuron culture and transfection

Hippocampal neurons were derived from embryonic day 18 fetal Sprague-Dawley rats and transfected at day in vitro 8 (DIV8) as previously described [27]. Briefly, dissociated hippocampal neurons were plated on poly-D-lysine coated glass coverslips and grown in the neurobasal medium supplemented with 2% B-27 (ThermoFisher scientific), 0.5 mM L-glutamine (Gibco) and 4 μM cytosine-1-β-d-arabinofuranoside (Ara-C; Sigma). At DIV8, neurons were transfected using the modified Ca^2+^ phosphate method. Briefly, 6 μg of DNA and 9.3 μl of 2 M CaCl_2_ were mixed in distilled water to a total volume of 75 μl, and the same volume of 2 × BBS [50 mM BES, 280 mM NaCl, and 1.5 mM Na_2_HPO_4_, pH 7.1] was added. The cell culture medium was completely replaced by transfection medium [minimum essential medium (MEM) 1 mM pyruvate, 0.6% glucose, 10 mM glutamine, and 10 mM HEPES, pH 7.65], and the DNA mixture was added to the cells and incubated in a 5% CO_2_ incubator for 60 min. Cells were washed twice with washing medium (pH 7.35) and then returned to the original culture medium. All animal experiments were approved by the Institute of Animal Care and Use Committee of Seoul National University, Korea.

### HEK293T cell transfection

HEK293T cells were transfected by Lipofectamine-2000 reagent (ThermoFisher scientific) following the manufacturer’s instruction. Briefly, 3 μg of plasmid DNA were mixed with 6 μl of Lipofectamine-2000 in the 200 μl of Opti-MEM solution (ThermoFisher scientific) followed by 20 min incubation at RT. Then, mixture was treated to HEK293T cells (60~ 70% confluency) in serum-free medium (Dulbecco’s Modified Eagle’s Medium, DMEM) for 3 h and the medium was replaced by complete medium (DMEM with 10% FBS). After 48 h, the cells were lysed for western blot.

### Immunocytochemistry

Cultured neurons were fixed in 4% paraformaldehyde in 4% sucrose/PBS for 15 min at RT and permeabilized with 0.25% triton X-100 solution for 5 min at RT. After permeabilization, neurons were blocked with 10% BSA/PBS for 30 min at RT. Then, neurons were incubated with primary antibody in 3% BSA/PBS for 2 h at RT and with Alexa Fluor conjugated secondary antibody in 3% BSA/PBS for 45 min at RT.

### FRAP assay

Cultured hippocampal neurons were transfected with GFP-synapsin and fluorescence recovery after photobleaching (FRAP) assay was performed on a Fluoview-1000 confocal microscope (Olympus) with a 100 x, 1.4 N.A. objective lens. Neurons were incubated in pre-warmed tyrode solution [136 mM NaCl, 2.5 mM KCl, 2 mM CaCl_2_, 1.3 mM MgCl_2_, 10 mM HEPES and 10 mM glucose], and single bouton was bleached to 50% of the original fluorescence intensity by scanning with a 488 nm laser at 50% of total laser power for 2 s. Time-lapse images were acquired every 5 s for 150 s and analyzed by using Olympus Fluoview software and OriginPro 9.0 (OriginLab).

### Synaptic vesicle tracking and analysis

GFP-synaptophysin expressing neurons were time-lapse imaged for 1 min with 0.5 s intervals to track the synaptic vesicle movements. Each x and y coordination of synaptic vesicles in time-lapse images was acquired using MetaMorph software (Molecular Devices) and the mean square displacement (MSD) was calculated using formula below [27, 52].

$$ \mathrm{MSD}\left(\mathrm{n}\uptau \right)=\frac{1}{N-n}{\sum}_{i=1}^{N-n}\left[{\left(x\left(\left(i+n\right)\tau \right)-x\left( i\tau \right)\right)}^2+{\left(y\left(\left(i+n\right)\tau \right)-y\left( i\tau \right)\right)}^2\right] $$

xi and yi are coordinates of synaptic vesicle, N is the total number of steps in the trajectory and *τ* is the acquisition time. First five points of the MSD versus time were linear-fitted and the diffusion coefficient was calculated using the equation MSD (nτ) ≈ 4*Dnτ*.

### Image acquisition and data analysis

Time-lapse images were acquired with an Olympus IX-71 inverted microscope (Olympus) with 40 x oil lens (1.0 N.A.) using an Andor iXon 897 EMCCD camera (Andor Technologies) and Touchbright LED light source (LCI) controlled by MetaMorph software. Tyrode solution included 10 μM 6-cyano-7-nitroquinoxaline-2,3-dione to prevent any recurrent excitation. Analysis and quantification of data were performed with MetaMorph software, ImageJ (NIH) and OriginPro 9.0 in a double*-* blind manner to avoid experimenter bias. Statistical comparisons were performed with Origin 9.0 and SPSS (IBM) software. Student’s *t* test was performed for comparisons between two independent groups. For multiple group comparison, one-way ANOVA followed by Tukey’s post hoc test was performed.

### Ca^2+^ measurements

To detect the Ca^2+^ dynamics, cultured neurons were incubated with 0.5 μM Fluo-4 AM Ca^2+^ indicator (F14201, ThermoFisher scientific) for 15 min at 37 °C. After 10 min of wash-out in tyrode, Fluo-4 AM intensity was measured before and after 5 min treatment of sAβ_1–42_. To see the blockage effect of TPP (Sigma), Fluo-4 AM loaded neurons were pre-incubated with extracellular buffer as indicated (normal tyrode (2 mM Ca^2+^), Ca^2+^-free tyrode containing EDTA, normal tyrode containing 2 μM TPP, Ca^2+^-free tyrode containing EDTA and 2 μM TPP) for 5 min and the changes in Fluo-4 AM intensity after sAβ_1–42_ or TPP treatment were measured by time-lapse imaging (5 s intervals). To confirm the levels of synapsin phosphorylation induced by sAβ_1–42_ in 0 mM extracellular Ca^2+^, neurons were each treated with DMSO, 2 μM TPP, 200 nM sAβ_1–42_, or 200 nM sAβ_1–42_ with 2 μM TPP for 2 h in Ca^2+^-free tyrode containing EDTA. After then, neuron lysates from each treatment group were western blotted to measure the level of phospho- and total-synapsin I.

### Immunoprecipitation

Transfected HEK293T cells were lysed in a 1% triton X-100 lysis buffer [20 mM Tris-HCl, pH 8, 1% triton X-100, 10% glycerol, 137 mM NaCl, 2 mM EDTA] with 1% seine/threonine phosphatase inhibitor (Sigma) and protease inhibitor cocktail (Sigma). After sonication and centrifugation, anti-synapsin I antibody (Synaptic Systems) was added to each of equal amounts of total cell lysate (500 μg). The samples were incubated overnight at 4 °C, then 30 μl Protein A-Sepharose (GE healthcare) was added and incubated for 1 h at 4 °C. The samples were washed three times with lysis buffer and then bead pellets were eluted with 30 μl of 2× sample buffer [100 mM Tris-HCl (pH 6.8), 4% sodium dodecyl sulfate, 0.2% bromophenol blue, 20% glycerol and 2% beta-mercaptoethanol] followed by boiling (100 °C, 5 min) and gel loading.

### Western blot

Cultured rat hippocampal neurons at DIV14–16 were lysed with 1% triton X-100 lysis buffer with 1% seine/threonine phosphatase inhibitor (Sigma) and protease inhibitor cocktail (Sigma). Lysates were centrifuged at 14,000 g at 4 °C for 20 min after sonication. Supernatants were collected and protein concentration was measured using BCA assay kit. Equal amounts of protein were loaded on to polyacrylamide gels. Gels were transferred to PVDF membranes (Pall Life Sciences, Ann Arbor, MI), then the membranes were incubated with 10% BSA/PBS or 5% SKIM milk/PBS for 30 min at RT. After washing in TBST, PVDF membranes were incubated with the primary antibody for overnight at 4 °C, followed by the horseradish peroxidase (HRP)-conjugated secondary antibody (Jackson ImmunoResearch Laboratories, West Grove, PA) for 1 h at RT. ECL solution (AbClon) and LAS 4000 (GE healthcare) were used to detect immunoreaction. Band intensities were calculated using imageJ.

## Additional file

Additional file 1: **Figure S1.** Phospho-deficient mutants of synapsin serine 9 (S9A) residue and actin binding. (**a**) Representative western blot images for immunoprecipitation and total cell lysate. (**b**) Quantitative analysis from 4 independent blots (1±0 for control, 1.94±0.27 for S9A). Values are means±SEM. N.S = no significant difference, * *p* < 0.05 (Student’s *t*-test). (PDF 345 kb)

## Abbreviations

A. F. U: Arbiturary fluorescence unit
AD: Alzheimer’s disease
Aβ: Amyloid beta
Ca^2+^: Calcium
CaMKIV: Ca^2+^/calmodulin-dependent protein kinases IV
Diffusion Coeffi: Diffusion coefficient
DIV: Day in vitro
F: Fluorescence
FCCP: Carbonyl cyanide p-(trifluoromethoxy)-phenylhydrazone
FRAP: Fluorescence recovery after photobleaching
IB: Immuno blot
MSD: Mean square displacement
P-Syn: Phospho-synapsin
RT: Room temperature
sAβ_1–42_: Prefibrillar form of soluble Aβ_1–42_
SV: Synaptic vesicle
Syn: Synapsin
TPP: Tetraphenylphosphonium
VDCC: Voltage dependent calcium channel
WT: Wild type
