# Supplementary figures and images for: Soluble Aβ1–42 increases the heterogeneity in synaptic vesicle pool size among synapses by suppressing intersynaptic vesicle sharing
Source: Mol Brain. 2018 Feb 20;11:10. doi: 10.1186/s13041-018-0353-z (PMC5819658; doi:10.1186/s13041-018-0353-z)

**a**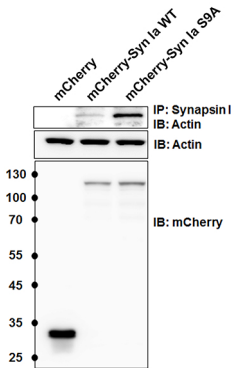**b**

Actin co-precipitated with Syn Ia

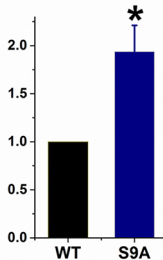

Supplement: Supplementary file 1 — Figure S1. Phospho-deficient mutants of synapsin serine 9 (S9A) residue and actin binding. (a) Representative western blot images for immunoprecipitation and total cell lysate. (b) Quantitative analysis from 4 independent blots (1±0 for control, 1.94±0.27 for S9A). Values are means±SEM. N.S = no significant difference, * p < 0.05 (Student’s t-test). (PDF 345 kb) [file 13041_2018_353_MOESM1_ESM.pdf]
